# Supplementary material for: Dielectric elastomer actuators based on stretchable and self-healable hydrogel electrodes
Source: R Soc Open Sci. 2019 Aug 7;6(8):182145. doi: 10.1098/rsos.182145 (PMC6731732; doi:10.1098/rsos.182145)
Supplement: Supplementary material from Dielectric Elastomer Actuators Based on Stretchable and Self-Healable Hydrogel Electrodes [file rsos182145supp1.pdf]

Electronic sumplementary materials for “Dielectric Elastomer Actuators Based on Stretchable and Self-Healable Hydrogel Electrodes”

Yang Gao, Xiaoliang Fang, Danhquang Tran, Kuan Ju, Bo Qian*, Jin Li*

[a]*School of Mechanical and Power Engineering, East China University of Science and Technology, Shanghai 200237 (China)*

Keywords: compliant electrodes, self-healing, CNT/PVA hydrogel

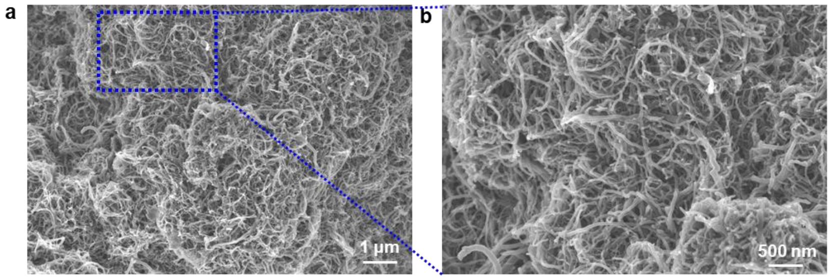

Figure S1. (a) SEM images of multi-wall CNTs. Multi-wall CNTs with an average diameter ~30 nm is bought from Shengzhen Nanotech Port Co. Ltd and used with no further purification. (b) Partial enlarged view of (a).

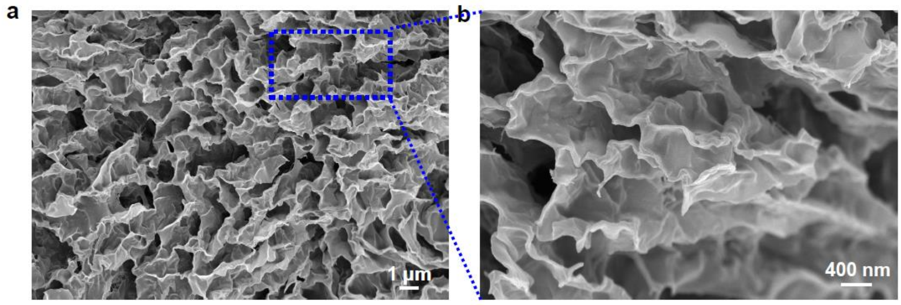

Figure S2. (a) SEM fractographic images of the hydrogel electrode sample based on the CNT/PVA after one-day freeze-dry. (b) Partial enlarged view of (a).

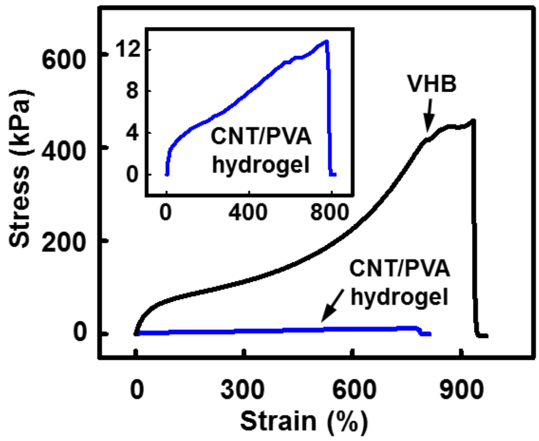

Figure S3. Stress – strain curves of the CNT/PVA hydrogel electrodes and VHB dielectric layer. Black curve shows stress-strain feature of the sample based on VHB and CNT/PVA hydrogel. It breaks down at ~900% strain with ~450 kPa. Blue curve in inset shows the details of stress-strain curve of the sample based on pure CNT/PVA hydrogel and it breaks down at ~800% stain with ~12 kPa.

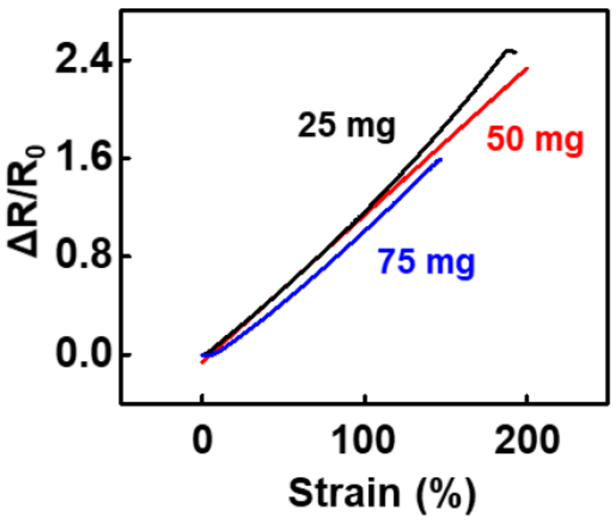

Figure S4. Relative resistance changes of the hydrogel electrodes prepared using 25 mg, 50 mg, and 75 mg of CNTs at strain ranging from 0% to 200%. Other materials used for the sample remains the same ratio as described in *Preparation of CNT/PVA hydrogel electrodes* section in the article.

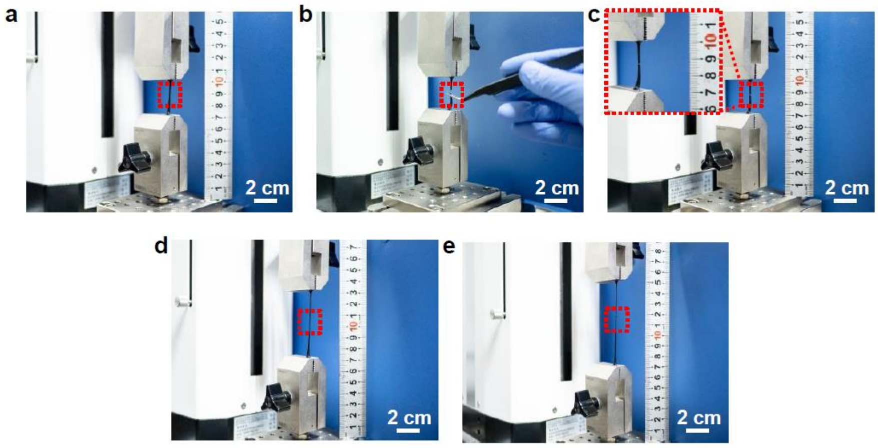

Figure S5. Sequential photographs showing the stretching test of CNT/PVA hydrogel electrode after a self-healing process. (a) Original state of CNT/PVA hydrogel electrode before the cutting. (b) CNT/PVA hydrogel electrode after the cutting. (c) Self-healing of the CNT/PVA hydrogel electrode. (d) Stretching of the electrode to the strain of ~100%. (e) Stretching of the electrode to the strain of ~170%.

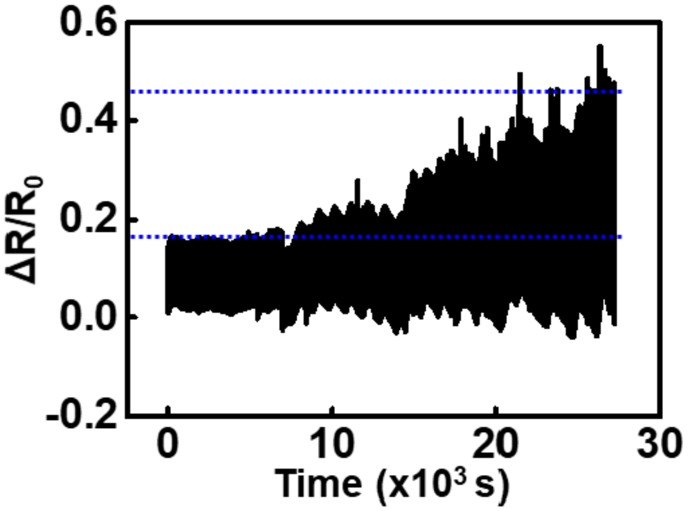

Figure S6. The stability test of the CNT/PVA hydrogel after cutting-self healing process. The CNT/PVA hydrogel electrode was tested at ~20% strain and last for ~28000s (~2000 cycles in total).
